# Clinical outcomes of unilateral biportal endoscopic discectomy vs. microdiscectomy in lumbar disc herniation

**DOI:** 10.3389/fmed.2026.1758130

**Published:** 2026-02-10

**Authors:** Yi He, Peng-fei Cao, Yin Zhang, Xun-an Xu, Tong-guang Xu

**Affiliations:** Department of Orthopedics, The People’s Hospital of Suzhou New District, Suzhou, China

**Keywords:** clinical effect, complications, lumbar disc herniation, microscopic nucleus pulposus removal, unilateral biportal endoscopic discectomy

## Abstract

**Background:**

Lumbar disc herniation (LDH) often causes radiculopathy, resulting in unilateral leg pain and lower back discomfort. Minimally invasive techniques such as microscopic discectomy (MSLD) and unilateral biportal endoscopic (UBE) discectomy are widely used. However, comparative data are limited. This study compares the clinical outcomes and complications of UBE and MSLD.

**Methods:**

A single-center, retrospective, non-randomized analysis was conducted on 80 patients with LDH who underwent surgical treatment at People’s Hospital of Suzhou New District between January 2021 and September 2023. Patients were categorized according to the surgical technique received into a microscope group (MG, *n =* 38) and endoscopic group (EG, *n =* 42). Pre- and postoperative pain (VAS), functional recovery (ODI, JOA), and intervertebral space height were assessed. Postoperative efficacy and complication rates were evaluated at a 6-month follow-up.

**Results:**

Compared with MG, the EG group was associated with shorter operation and hospitalization times and less intraoperative blood loss (*p =* 0.03, *p =* 0.02). VAS, ODI, and JOA scores improved over time in both groups, with greater early improvements observed in EG at 3 days, 3 months, and 6 months postoperatively (*p =* 0.04, *p =* 0.01, *p =* 0.02). At 6 months, EG was also associated with better preservation of intervertebral space height (*p =* 0.04). No statistically significant differences were observed between groups in terms of overall clinical efficacy or postoperative complication rates (*p* > 0.05).

**Conclusion:**

Both UBE and MSLD are effective surgical options for the treatment of LDH. The findings suggest that UBE may offer advantages in surgical efficiency, early pain relief, functional recovery, and intervertebral space preservation. Given the retrospective, non-randomized design, selection bias and residual confounding cannot be entirely excluded, so these associative findings require confirmation in prospective studies with longer follow-up.

## 1 Introduction

Lumbar disc herniation (LDH) is among the common diseases in spinal surgery, frequently resulting in sciatica and low back pain. In severe cases, compression of the cauda equina may occur, leading to significant neurological deficits and a substantial decline in patients’ quality of life (1, 2). In clinical practice, conservative treatment is typically the first-line approach for managing LDH. However, surgical intervention becomes necessary when conservative treatments fail or if there is progressive deterioration of symptoms. Traditional lumbar discectomy, while effective, has several drawbacks including a large incision, reduced stability of the iatrogenic lumbar vertebra, and potential complications such as nerve root adhesion. These factors can prolong hospitalization and increase healthcare costs (3, 4). Recent advances in minimally invasive techniques and spinal instrumentation, microscopic and endoscopic approaches have been increasingly adopted in the surgical management of LDH. Microscopic discectomy (MSLD) provides a three-dimensional surgical field, enhances visualization of deep anatomical structures, and improves identification of pathological targets while minimizing injury to adjacent neural tissues during surgery (5, 6). Nevertheless, MSLD typically requires laminar resection, paraspinal muscle stripping, and partial bone removal, which may compromise the stability of the posterior spinal elements. To reduce the influence on the posterior structure of the spine, endoscopy is gradually used in the field of spinal diseases. Unilateral biportal endoscopic (UBE) discectomy via posterior intervertebral plate approach can achieve central spinal canal decompression on the one hand and reduce the pressure of bilateral nerve roots and lateral recess on the other hand, which provides a new choice for LDH treatment. Unilateral biportal endoscopic (UBE) discectomy via the posterior intervertebral plate approach can achieve central spinal canal decompression on the one hand and reduce the pressure of bilateral nerve roots and lateral recess on the other hand, offering a promising option for LDH treatment. While clinical practice predominantly focuses on evaluating the individual efficacy of UBE and MSLD, there remains a dearth of studies that compare both techniques in a standardized manner. Our study aims to address this gap by focusing on the comparative clinical outcomes of UBE and MSLD, especially concerning early postoperative recovery and intervertebral space preservation. We hypothesized that UBE would result in faster recovery and better structural preservation compared to MSLD.

While previous studies have shown comparable outcomes in terms of overall efficacy, this research provides a detailed evaluation of both early and intermediate postoperative intervals, specifically addressing the important aspect of intervertebral space preservation a factor often overlooked in prior studies. Thus, this study not only compares clinical effectiveness and complications but also offers insights into the nuances of early functional recovery and anatomical preservation. Our findings provide new evidence on the efficacy and safety profiles of these two minimally invasive procedures, contributing valuable data for clinical decision-making.

## 2 Materials and methods

### 2.1 Research flow chart

**Figure 1:**
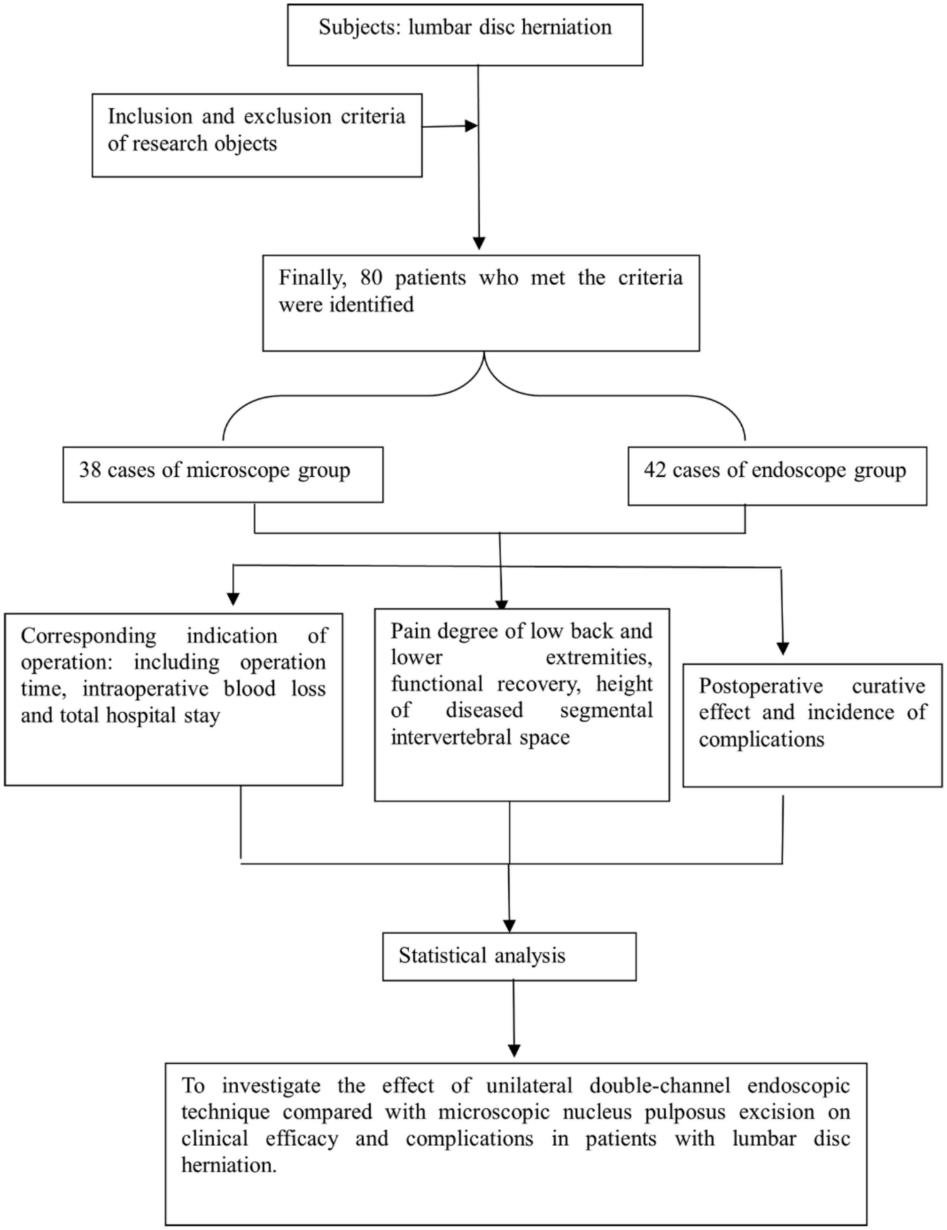
Research flow chart.

Figure 1 shows the flow chart of this research.

### 2.2 Research design

This retrospective single-center study included a total of 80 patients diagnosed with LDH who underwent surgery at the People’s Hospital of Suzhou New District, between January 2021 and September 2023. Of these, 38 patients had previously received MSLD and were categorized into the microscope group (MG), while 42 patients had undergone UBE and were categorized into the endoscope group (EG). Grouping was therefore based on the surgical technique received. We prepared this manuscript in accordance with the STROBE (Strengthening the Reporting of Observational Studies in Epidemiology) checklist for observational studies (Supplementary file S1).

The choice of surgical modality (UBE vs. MSLD) was made through shared decision-making, considering anatomical feasibility and patient preference. Both approaches were regarded as technically suitable for single-level LDH with imaging-confirmed nerve root compression and failed conservative management. All procedures were performed by the same experienced surgical team.

All patients were rigorously screened according to strict inclusion and exclusion criteria to ensure cohort uniformity and data integrity. Inclusion criteria were as follows: (1) meeting the diagnostic criteria for LDH, characterized by radiating pain or numbness in the lower extremities, with or without accompanying low back pain, and with corresponding sensory and myodynamia deficits in the lower limbs (7); (2) Presence of single-segment LDH confirmed via imaging modalities such as CT and MRI, with clinical symptoms consistent with imaging findings; (3) lack of symptom improvement following conservative treatment for more than 90 days; (4) adherence to relevant surgical indications (8, 9), with surgical intervention conducted for the first time at our hospital; and (5) a minimum follow-up duration of ≥ 6 months, having all clinical data at hand. Exclusion criteria included: (1) individuals with lumbar spondylolisthesis and compromised lumbar stability; (2) individuals with multiple-segment LDH; (3) individuals with a history of previous lumbar surgery; (4) individuals presenting with other spinal pathologies such as cauda equina syndrome, ankylosing spondylitis, spinal deformity, spinal tumor, or spinal tuberculosis; and (5) those with lumbar fractures. Additionally, patients with severe underlying medical conditions deemed unfit for anesthesia or surgery, as well as those with mental illness or cognitive impairment, were not included in the study. The study was approved by the Ethics Committee of the People’s Hospital of Suzhou New District. The need for individual informed consent was waived due to the retrospective design.

### 2.3 Research object

#### 2.3.1 General information

In the microscopic group, a total of 38 patients were enrolled, with ages ranging from 41 to 72 years and a mean age of 50.29 ± 5.39 years. Among them, 21 patients (55.26%) were male and 17 (44.74%) were female. The body mass index (BMI) ranged from 21.29 to 25.51 kg/m^2^, with an average BMI of 23.82 ± 1.56 kg/m^2^. Additionally, 12 patients (31.58%) reported smoking, and 9 (23.68%) reported alcohol consumption. Comorbidities included diabetes mellitus in 12 patients (31.58%) and hypertension in 9 patients (23.68%). Two patients (5.26%) had a prior history of lumbar sprain. The duration of symptoms ranged from 3 to 23 months, with a mean duration of 7.72 ± 1.28 months. The distribution of lesion segments was as follows: L3/4: 4 cases (10.53%), L4/5: 21 cases (55.26%), and L5/S1: 13 cases (34.21%). Regarding education background, 20 patients (52.63%) had completed primary or junior high school, 10 (26.32%) had completed technical secondary or senior high school, and 8 (21.05%) had attained a college-level education or higher.

In the endoscopic group (EG), 42 patients were aged between 41 and 70 years, with a mean age of 50.32 ± 5.43 years. Of these, 24 patients (57.14%) were male and 18 (42.86%) were female. The BMI ranged from 21.30 to 25.67 kg/m^2^, with a mean BMI of 23.79 ± 1.47 kg/m^2^. Ten patients (23.91%) reported smoking, and 13 (30.95%) reported alcohol consumption. Comorbidities included diabetes mellitus in 9 patients (21.43%) and hypertension in 11 patients (26.19%). Three patients (7.14%) had a history of lumbar sprain. The duration of disease ranged from 2.5 to 21 months, with a mean duration of 7.69 ± 1.26 months. This prolonged duration aligns with clinical practice in resource-limited settings, where patients often face delays in surgical referral due to extended conservative management, limited access to specialized care, or reluctance to pursue surgery (10). Moreover, the distribution of lesion segments was as follows: L3/4: 3 cases (7.14%), L4/5: 25 cases (59.52%), and L5/S1: 14 cases (33.33%). Regarding education level, 22 patients (52.38%) had completed primary and middle schools, 11 (26.19%) completed senior high schools and secondary schools, and 9 (21.43%) had attained a junior college education or higher. There were no statistically significant differences in baseline characteristics, including age, sex, BMI, comorbidities, symptom duration, lesion level, or educational background, between the two groups (*p* > 0.05), indicating good comparability.

### 2.4 Methods

#### 2.4.1 MSLD is used in the MG

Patients in the MSLD group underwent standard single-level microdiscectomy under general anesthesia. After localization of the target segment, a limited laminotomy and flavectomy were performed under microscopic visualization to decompress the affected nerve root. The procedure followed widely accepted microsurgical principles without modification and was performed by the same surgical team in all cases, ensuring procedural consistency. Closed-suction drains were placed routinely in all cases (UBE and MD) and removed per unit protocol at <50 mL over the preceding 24 h with stable neurology (11).

#### 2.4.2 UBE was used in the endoscope group

Following preoperative preparation, patients were placed in the prone position after induction of general anesthesia, and vital signs were monitored throughout the procedure. The affected intervertebral space was localized using fluoroscopy. Two small transverse incisions were made approximately 1.5 cm apart, just off the midline. The endoscopic working portal was positioned at the lower edge of the upper lamina, and the instrumentation portal was placed at the upper edge of the lower lamina. The endoscopic entry channel was irrigated with normal saline to ensure a clear visual field. Subsequently, soft tissue found on the outside of the vertebral lamina was meticulously removed using an electrocoagulation knife to control bleeding. In order to remove the ligamentum flavum and completely clean the top and lower edges of the vertebral lamina, rondeurs and high-speed drills were used. Careful manipulation allowed for adequate exposure and protection of the nerve root and dural sac, facilitating exploration of the spinal canal to identify the protruding nucleus pulposus within the affected segment. Adhesions were released, and a retractor was utilized to safeguard the dural sac during nucleus pulposus removal. Upon completion, the endoscope and instruments were withdrawn, and each incision was closed with a single suture.

#### 2.4.3 Intraoperative blood loss

Intraoperative blood loss was calculated by measuring the volume of blood collected in the suction canister during surgery, as well as by accounting for any blood-soaked sponges used during the procedure. The total blood loss was determined by adding the measured blood collected in the canisters and the blood volume absorbed by the sponges, with the latter estimated based on the weight of the sponges and the known absorption capacity. All measurements were taken immediately after the completion of each procedure to ensure accuracy.

#### 2.4.4 Postoperative treatment

All patients followed a standardized routine care per institutional order. Charts showed no systematic differences between groups. Usual pain control was paracetamol plus an NSAID/COX-2, with short-course opioid rescue if needed; no epidural or local steroid injections were used. Drains were removed when output was <50 mL in the prior 24 h or at 48 h, whichever came first. Mobilization began post-op day 1 (sit/stand), with assisted walking by day 3 and supervised core/back exercises from day 7. Sutures were removed on days 12–14. Patients were advised to avoid heavy lifting/strenuous activity for 12 weeks. Nursing, physiotherapy, and discharge criteria were uniform across cohorts and throughout the study period.

#### 2.4.5 Surgeon experience

Surgeon experience was controlled by ensuring that all surgeries in this study were performed by a team with standardized training and technique. To account for potential variations in surgical proficiency, the surgeons’ experience with the procedures was assessed by the total number of cases they had performed prior to the study. The lead surgeon had performed more than 100 cases of each procedure (UBE and MSLD), while the assisting surgeons had completed at least 50 cases in total, with a minimum of 20 cases for each technique. This information was recorded and used to ensure that all procedures were carried out by surgeons with adequate experience.

### 2.5 Observation index

All patient were followed up for at least 6 months, during which the following outcomes were assessed:

1. Indications for operation: including total hospitalization time, intraoperative blood loss and operation time.

Operation time is the duration from the first skin incision to the last skin suture. Intraoperative blood loss was recorded from anesthesia records and operative notes, calculated as (suction canister volume − total irrigation) + net gauze weight (1 g = 1 mL). Total hospitalization time denotes the period from admission to discharge.

1. Pain intensity.

Assessed using the Visual Analogue Scale (VAS) for low back and lower limb pain (12). The VAS consists of a 10-cm horizontal line, with 0 cm representing “no pain” and 10 cm representing “worst possible pain.” Patients marked their current pain level on the line, and the distance from 0 cm to the mark was measured in centimeters (range: 0–10). Assessments were conducted preoperatively and at 3 days, 3 months, and 6 months postoperatively.

1. Functional recovery.

The Oswestry Disability Index (ODI): A 10-item questionnaire evaluating disability related to low back pain. Each item is scored from 0 to 5, yielding a total raw score ranging from 0 to 45. The ODI score was calculated as (total raw score / 50) × 100%, resulting in a percentage from 0% (no disability) to 100% (maximum disability) (13). The nine items assess the following domains: pain severity, ability to perform self-care activities in daily life, standing ability, sitting comfort, walking ability, lifting ability, quality of sleep, impact on sexual life, and participation in social activities.

Japanese Orthopaedic Association (JOA) score: A 29-point scale assessing subjective symptoms (9 points), clinical signs (6 points), and activities of daily living (14 points). Higher scores indicate better function (14).

1. Intervertebral space height.

The intervertebral space heights of the two groups were compared before operation, 3 days, 3 months, and 6 months after operation. Lumbar lateral CT examination was performed at the anterior, posterior, and central position of the vertebral body. The average disc height was calculated as [(anterior height + middle height + posterior height) / 3] in millimeters (mm).

1. Postoperative curative effect.

At the final follow-up, which occurred 6 months post-operation, the modified MacNab score (15) was employed to assess the surgical outcomes of the patients. This scoring system categorizes outcomes as follows:

Excellent: Patients resumed a normal quality of life, with complete relief of symptoms.

Good: Although clinical manifestations and signs of LDH persist, patients were able to independently carry out their daily activities and work.

Fair: Definite subjective improvement but with significant limitations.

Poor: No improvement or worsening of symptoms. Excellent and good rate = (number of excellent cases + good cases) / total number of cases × 100%.

1. Complications.

The complication were recorded within six-month postoperatively, including dural tears (16), poor wound healing (17), lumbar instability (18), muscle hematomas (19), radicular pain (20), and other adverse events. The incidence of complications = the total number of cases / total cases of various complications × 100%.

### 2.6 Blinded radiographic measurements

Radiographic measurements of intervertebral space height were performed by two independent assessors who were blinded to the surgical technique used for each patient. Both assessors were experienced radiologists with expertise in spinal imaging. The measurements were taken from postoperative X-rays obtained at the 6-month follow-up, and the average of the two assessors’ readings was used for final analysis to reduce any measurement bias.

### 2.7 Statistical method

Data were analyzed using SPSS 22.0 (IBM Corp., Armonk, NY, USA), and figures were generated using Prism 9.4.1 (GraphPad Software, San Diego, CA, USA). Continuous data (e.g., VAS, ODI, JOA, intervertebral space height) were expressed as mean ± standard deviation (SD) and assumed to follow a normal distribution with homogeneous variance. Repeated-measures analysis of variance (RM-ANOVA) was used to assess the effects of time, group (Endoscopic vs. Microscopic Discectomy), and time × group interaction on continuous outcomes. *Post-hoc* Tukey tests were performed to compare groups at each time point (pre-operative, 3 days, 3 months, 6 months post-operative), with multiplicity adjustment to control the family-wise error rate. Partial *η*^2^ was calculated to estimate effect sizes for RM-ANOVA interactions. Categorical data (e.g., MacNab scores, complications) were expressed as counts and percentages [*n* (%)] and compared using the *χ*^2^ test. A *p* <0.05 was considered statistically significant.

## 3 Results

### 3.1 Demographic and baseline characteristics of patients

Table 1 summarizes the demographic and baseline clinical characteristics of patients in both groups. There were no statistically significant differences between the UBE and MSLD groups in terms of age, sex distribution, BMI, smoking or alcohol use, comorbidities (diabetes mellitus and hypertension), lumbar sprain history, disease duration, or the distribution of herniation segments (L3–L4, L4–L5, L5–S1). Education levels were also comparable between groups. All *p*-values exceeded 0.05, confirming baseline comparability and minimizing selection bias.

**Table 1:** Patient demographic and clinical characteristics.

Characteristics	Microscopic (*n =* 38)	Endoscopic (*n =* 42)	t/*χ*^2^	*P*-value
Age (years)	50.29 ± 5.39	50.32 ± 5.43	t = −0.025	0.980
Gender			*χ*^2^ = 0.000	1.000
Male	21 (55.26%)	24 (57.14%)		
Female	17 (44.74%)	18 (42.86%)		
BMI (kg/m^2^)	23.82 ± 1.56	23.79 ± 1.47	t = 0.088	0.930
Smoking	12 (31.58%)	10 (23.91%)	*χ*^2^ = 0.277	0.599
Alcohol consumption	9 (23.68%)	13 (30.95%)	*χ*^2^ = 0.227	0.634
Comorbidities				
Diabetes mellitus	12 (31.58%)	9 (21.43%)	*χ*^2^ = 0.602	0.438
Hypertension	9 (23.68%)	11 (26.19%)	*χ*^2^ = 0.000	1.000
Lumbar sprain history	2 (5.26%)	3 (7.14%)	*χ*^2^ = 0.000	1.000
Disease duration (months)	7.72 ± 1.28	7.69 ± 1.26	t = 0.105	0.916
Lesion segments			*χ*^2^ = 0.329	0.849
L3–L4	4 (10.53%)	3 (7.14%)		
L4–L5	21 (55.26%)	25 (59.52%)		
L5–S1	13 (34.21%)	14 (33.33%)		
Education level			*χ*^2^ = 0.002	0.999
Primary and junior high	20 (52.63%)	22 (52.38%)		
Secondary and senior high	10 (26.32%)	11 (26.19%)		
College or higher	8 (21.05%)	9 (21.43%)		

Data presented as mean ± SD or number (%).

### 3.2 Comparison of corresponding indications

**Figure 2:**
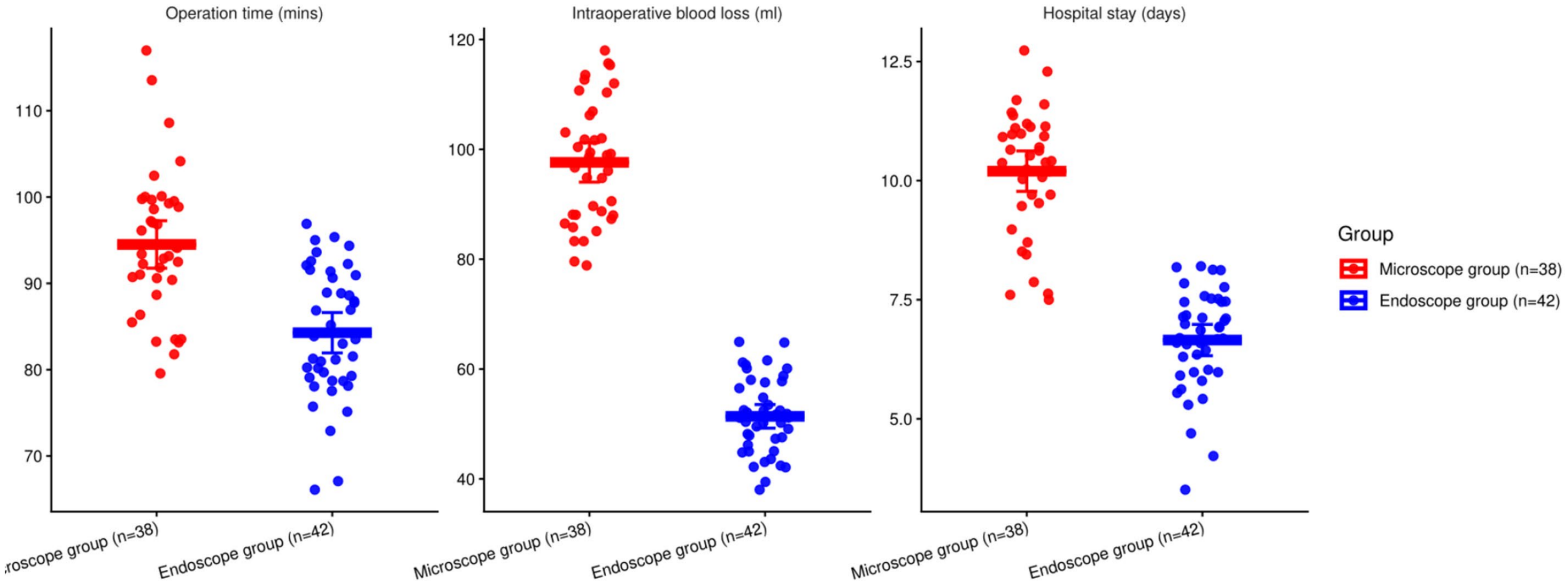
Comparison of corresponding indications involving the two groupings (*p < 0.05).

Compared with MSLD, UBE had shorter operative time (mean difference −9.61 min; 95% CI − 13.02 to −6.20; *p <* 0.001), lower blood loss (−46.58 mL; 95% CI − 50.74 to −42.42; *p <* 0.001), and shorter hospital stay (−3.04 days; 95% CI − 3.58 to −2.50; *p <* 0.001; Table 2 and Figure 2).

**Table 2:** Comparison of corresponding indications.

Outcome	Group	Mean ± SD	95% CI	*t*	*P*-value
Operation time (mins)	Microscope group (*n =* 38)	93.82 ± 8.12	91.15–96.49	5.542	<0.001
	Endoscope group (*n =* 42)	84.21 ± 7.39	81.91–86.51		
Intraoperative blood loss (ml)	Microscope group (*n =* 38)	99.45 ± 10.81	95.90–103.00	22.316	<0.001
	Endoscope group (*n =* 42)	52.87 ± 7.74	50.46–55.28		
Hospital stay (days)	Microscope group (*n =* 38)	9.83 ± 1.29	9.41–10.25	9.495	<0.001
	Endoscope group (*n =* 42)	6.79 ± 1.15	6.43–7.15		

### 3.3 Comparison of the degree of low back and lower limb pain

**Figure 3:**
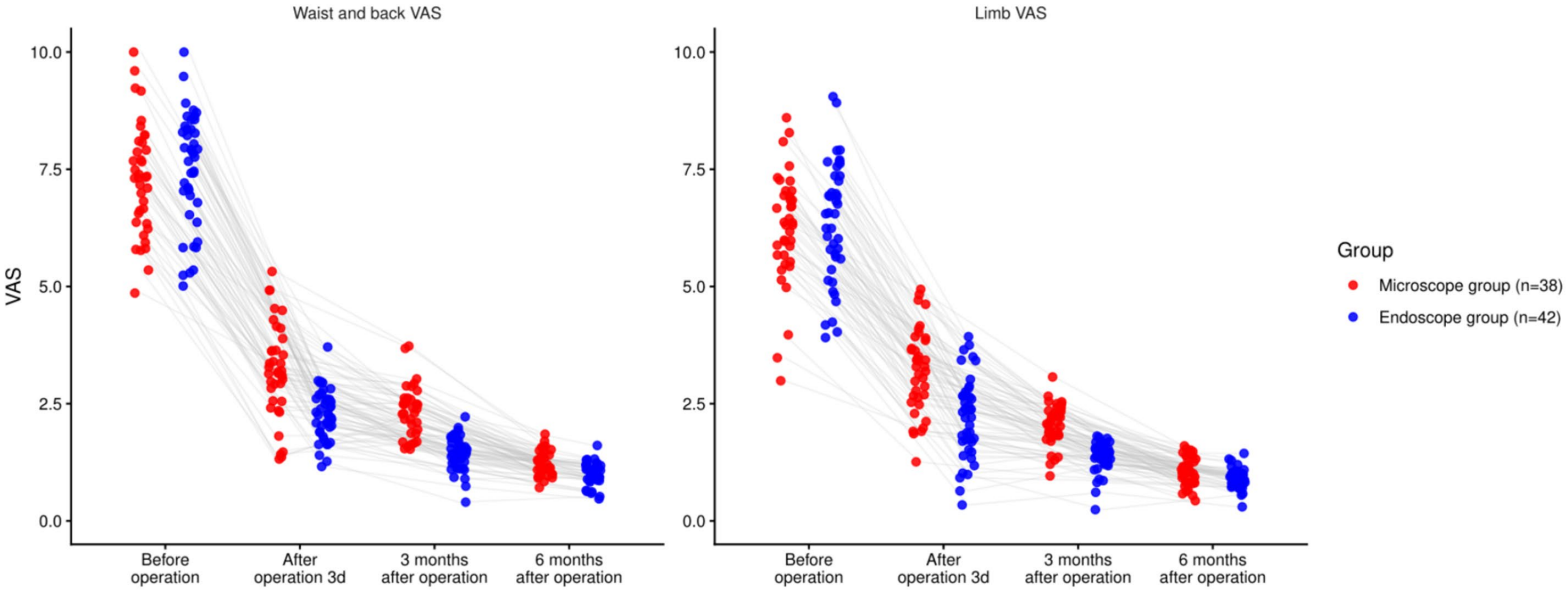
Comparison of pain degree of lower back and lower limbs involving the two groupings (^ns^*P >* 0.05, ^*^p < 0.05).

There were statistically significant effects of time, group, and time × group interaction on the VAS scores for both lower back and lower limb pain (*p <* 0.05). Baseline differences were non-significant (back *p =* 0.941; leg *p =* 0.908). At 3 days and 3 months, EG reported lower VAS than MG for both back and leg pain (all *p <* 0.001). At 6 months, EG remained lower for back pain (*p <* 0.001) and leg pain (*p =* 0.0019). However, the time × group interaction effect was non-significant due to the absence of a consistent trend across all time points. While the EG group showed greater pain relief early on, the interaction between time and group was not significant across the entire follow-up period. These findings are presented in Table 3 and Figure 3.

**Table 3:** Comparison of the degree of low back and lower limb pain.

Outcome	Time point	Group	Mean ± SD	95% CI
Waist and back VAS	Before operation	Microscope (*n =* 38)	7.21 ± 1.20	6.82–7.60
		Endoscope (*n =* 42)	7.19 ± 1.22	6.81–7.57
	After operation 3d	Microscope (*n =* 38)	3.16 ± 0.92	2.86–3.46
		Endoscope (*n =* 42)	2.14 ± 0.53	1.98–2.30
	3 months after operation	Microscope (*n =* 38)	2.25 ± 0.55	2.07–2.43
		Endoscope (*n =* 42)	1.42 ± 0.37	1.30–1.54
	6 months after operation	Microscope (*n =* 38)	1.27 ± 0.31	1.17–1.37
		Endoscope (*n =* 42)	0.98 ± 0.22	0.91–1.05
	*F*_time_/*P*	88.671/<0.001		
	*F*_Intergroup_/*P*	1.151/0.287		
	*F*_Time × intergroup_/*P*	1.632/0.183		
Limb VAS	Before operation	Microscope (*n =* 38)	6.32 ± 1.16	5.94–6.70
		Endoscope (*n =* 42)	6.29 ± 1.14	5.92–6.66
	After operation 3d	Microscope	3.27 ± 0.96	2.95–3.59
		Endoscope	2.24 ± 0.85	1.98–2.50
	3 months after operation	Microscope	2.10 ± 0.47	1.95–2.25
		Endoscope	1.36 ± 0.32	1.26–1.46
	6 months after operation	Microscope	1.05 ± 0.29	0.96–1.14
		Endoscope	0.87 ± 0.19	0.81–0.93
	*F*_time_/*P*	85.571/<0.001		
	*F*_Intergroup_/*P*	1.252/0.267		
	*F*_Time × intergroup_/*P*	1.845/0.140		

Notes: Data are mean ± SD. Per-timepoint EG vs MG contrasts may be shown for descriptive context but do not override the non-significant omnibus Group and Time × Group tests. RM-ANOVA summary (Waist/Back VAS): Time: F, partial *η*^2^ = 0.532; Group: F, partial *η*^2^ = 0.015; Time × Group: F, partial *η*^2^ = 0.020. RM-ANOVA summary (Limb VAS): Time: F, partial *η*^2^ = 0.523; Group: F, partial *η*^2^ = 0.016; Time×Group: F, partial *η*^2^ = 0.023.

### 3.4 Comparison of functional recovery

**Figure 4:**
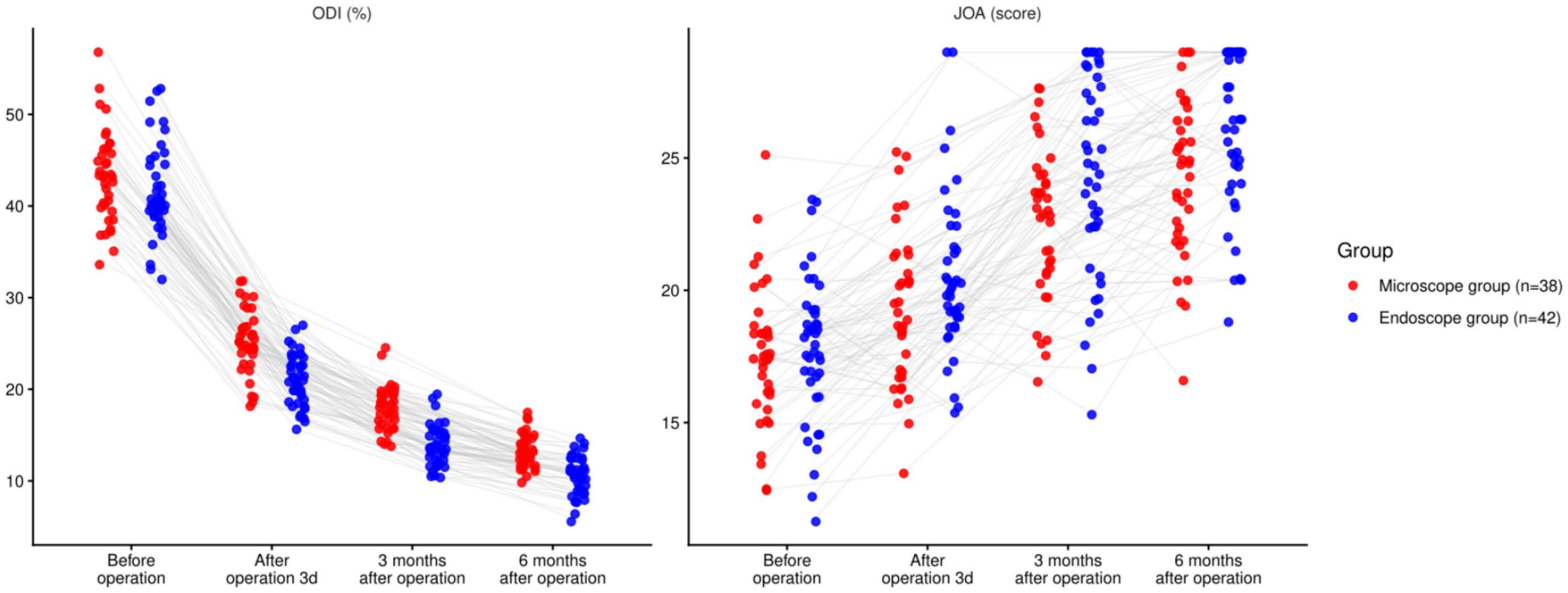
Comparison of functional recovery between two groups (^ns^*P >* 0.05, ^*^p < 0.05).

There was a significant time effect for both ODI and JOA (both *p <* 0.001). The group main effect (ODI: *p =* 0.095; JOA: *p =* 0.064) and the time × group interaction (ODI: *p =* 0.295; JOA: *p =* 0.299) were not significant. Baseline values did not differ (ODI *p =* 0.964; JOA *p =* 0.949). Exploratory between-group contrasts suggested lower ODI and higher JOA in the endoscope group at early timepoints, and at 6 months the endoscope group remained lower for ODI (−2.23 points; 95% CI − 3.13 to −1.33) and higher for JOA (+2.44 points; 95% CI + 0.94 to +3.94). These contrasts are presented for descriptive context and do not override the non-significant omnibus Group and Interaction tests (*p <* 0.05, Table 4; Figure 4).

**Table 4:** Comparison of functional recovery.

Outcome	Time point	Group	Mean ± SD	95% CI
ODI (%)	Before operation	Microscope (*n =* 38)	42.79 ± 4.87	41.22–44.36
		Endoscope (*n =* 42)	42.84 ± 4.89	41.29–44.39
	After operation 3d	Microscope (*n =* 38)	25.18 ± 3.14	24.13–26.23
		Endoscope (*n =* 42)	21.34 ± 2.97	20.42–22.26
	3 months after operation	Microscope (*n =* 38)	17.37 ± 2.39	16.58–18.16
		Endoscope (*n =* 42)	14.24 ± 2.11	13.59–14.89
	6 months after operation	Microscope (*n =* 38)	13.47 ± 2.14	12.78–14.16
		Endoscope (*n =* 42)	11.24 ± 1.97	10.63–11.85
	F _time_/P	91.340/<0.001		
	F _Intergroup_/P	2.859/0.095		
	F _Time × intergroup_/P	1.242/0.295		
JOA score	Before operation	Microscope (*n =* 38)	17.27 ± 2.75	16.38–18.16
		Endoscope (*n =* 42)	17.31 ± 2.79	16.45–18.17
	After operation 3d	Microscope (*n =* 38)	19.14 ± 2.87	18.24–20.04
		Endoscope (*n =* 42)	20.94 ± 3.02	20.00–21.88
	3 months after operation	Microscope (*n =* 38)	22.45 ± 3.11	21.42–23.48
		Endoscope (*n =* 42)	24.87 ± 3.49	23.77–25.97
	6 months after operation	Microscope (*n =* 38)	24.31 ± 3.32	23.25–25.37
		Endoscope (*n =* 42)	26.75 ± 3.54	25.64–27.86
	F _time_/P	49.080/<0.001		
	F _Intergroup_/P	3.520/0.064		
	F _Time × intergroup_/P	1.232/0.299		

Data are mean ± SD. Baseline EG vs MG: ODI *P =* 0.964; JOA *p =* 0.949. Exploratory per-timepoint contrasts (e.g., 6-month ODI Δ = −2.23, 95% CI −3.13 to −1.33; JOA Δ = +2.44, 95% CI +0.94 to +3.94). RM-ANOVA summary (ODI): Time: F, partial *η*^2^ = 0.539; Group: F, partial *η*^2^ = 0.035; Time × Group: F, partial *η*^2^ = 0.016; RM-ANOVA summary (JOA): Time: F, partial *η*^2^ = 0.386; Group: F, partial *η*^2^ = 0.043; Time × Group: F, partial *η*^2^ = 0.016.

### 3.5 Comparison of intervertebral space height

**Figure 5:**
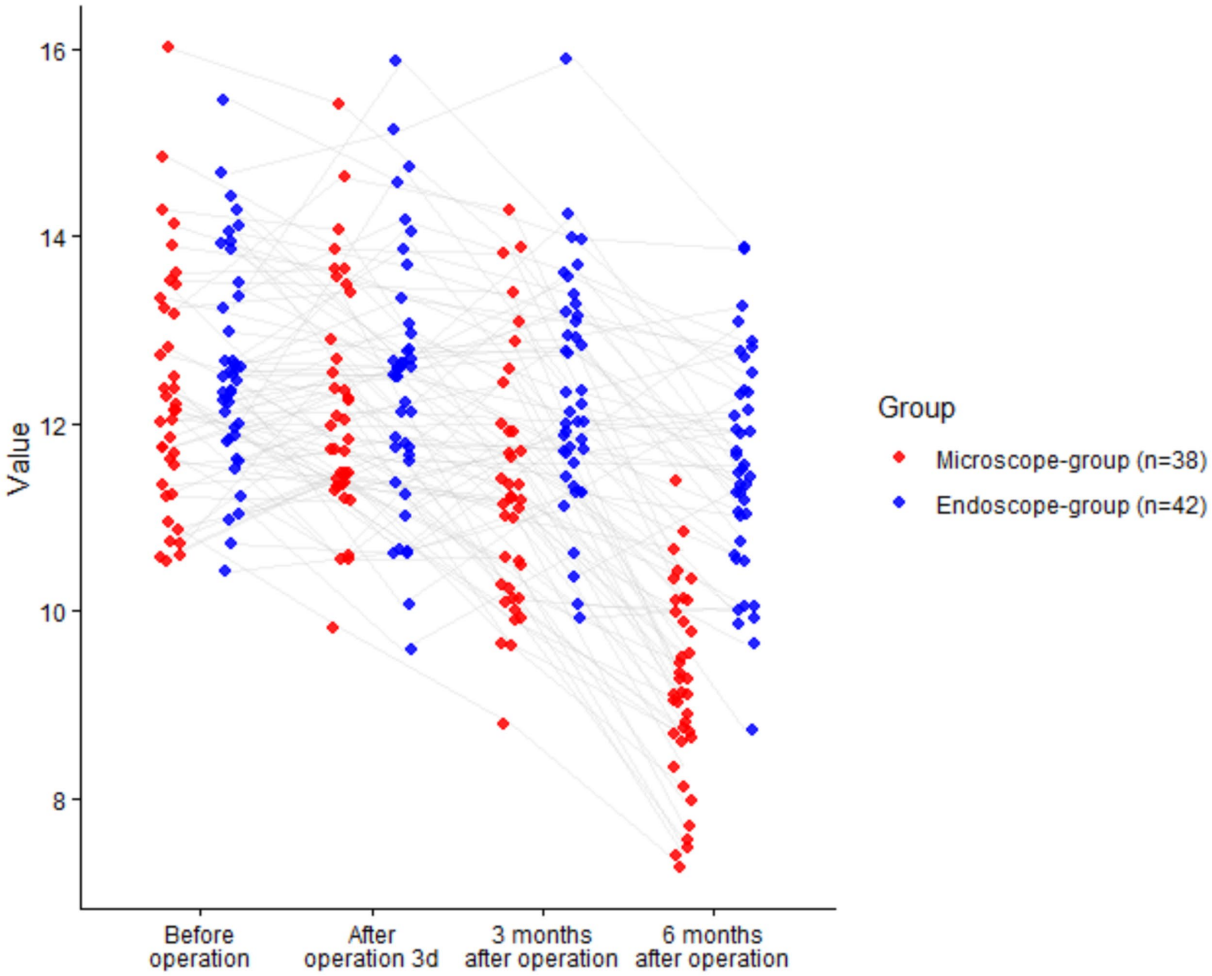
Comparison of intervertebral space height between two groups (^ns^*P >* >0.05, ^*^p < 0.05).

There was a significant time effect (*F* = 32.860, *p <* 0.001) and a significant time × group interaction (*F* = 9.183, *p <* 0.001), whereas the group main effect was not significant (*F* = 1.448, *p =* 0.232). Between groups, no differences were observed at baseline (*p =* 0.885), 3 days (*p =* 0.941), or 3 months (*p =* 0.116). At 6 months, disc height was better preserved with UBE (difference +1.96 mm; 95% CI + 1.47 to +2.45; *p <* 0.001), while both groups showed a decline from baseline over time (Table 5; Figure 5).

**Table 5:** Comparison of the height of diseased segmental intervertebral space between two groups (mm).

Time point	Group	Mean ± SD	95% CI
Before operation	Endoscope (*n =* 42)	12.52 ± 1.24	12.13–12.91
	Microscope (*n =* 38)	12.48 ± 1.23	12.07–12.89
After operation 3d	Endoscope (*n =* 42)	12.33 ± 1.21	11.95–12.71
	Microscope (*n =* 38)	12.31 ± 1.20	11.90–12.72
3 months after operation	Endoscope (*n =* 42)	12.01 ± 1.19	11.63–12.39
	Microscope (*n =* 38)	11.59 ± 1.17	11.20–11.98
6 months after operation	Endoscope (*n =* 42)	11.24 ± 1.15	10.88–11.60
	Microscope (*n =* 38)	9.28 ± 1.07	8.94–9.62
F _time_/P	32.860/<0.001		
F _Intergroup_/P	1.448/0.232		
F _Time × intergroup_/P	9.183/<0.001		

Data are mean ± SD. *Post-hoc* Tukey tests compared Endoscopic Group (EG) vs. Microscopic Discectomy (MG) at each time point, with multiplicity adjustment. RM-ANOVA summary: Time: F, partial *η*^2^ = 0.296; Group: F, partial *η*^2^ = 0.018; Time×Group: F, partial *η*^2^ = 0.105.

### 3.6 Comparison of postoperative curative effect

The proportion of patients achieving excellent or good outcomes based on the modified MacNab criteria was higher in the UBE group than in the MSLD group; however, the difference did not reach statistical significance (*p* > 0.05, Table 6).

**Table 6:** Comparison of postoperative curative effect.

Group	Excellent	Good	General	Poor	Excellent and good rate
Microscope group (*n =* 38)	19 (50.00)	12 (31.58)	6 (15.79)	1 (2.63)	31 (81.58)
Endoscope group (*n =* 42)	25 (59.52)	12 (28.57)	5 (11.90)	0 (0.00)	37 (88.09)

Statistical test: *χ*^2^ = 0.664, *p =* 0.415. Effect size: Risk difference (EG vs. MG) = 6.5% (95% CI: −9.2 to 22.3%); Risk ratio = 1.08 (95% CI: 0.90–1.30). Fisher’s exact test gave similar results (*p =* 0.535).

### 3.7 Comparison of complications

There wasn’t a noticeable variation in the incidence of postoperative complications involving the two groupings (*p* > 0.05), as shown in Table 7.

**Table 7:** Comparison of complications.

Complication	Microscope group (*n =* 38)	Endoscope group (*n =* 42)
Dural tear	0 (0.00%)	1 (2.38%)
Lumbar instability	1 (2.63%)	0 (0.00%)
Muscle hematoma	1 (2.63%)	0 (0.00%)
Radiation pain of affected limb	1 (2.63%)	1 (2.38%)
Poor wound healing	1 (2.63%)	0 (0.00%)
Total complications	4 (10.53%)	2 (4.76%)

Statistical test: *χ*^2^ = 0.956, *p =* 0.328. Effect size: Risk difference (EG vs. MG) = −5.8% (95% CI: −17.5 to 5.9%); Risk ratio = 0.45 (95% CI: 0.09–2.33). Due to small cell counts, Fisher’s exact test was also performed (*p =* 0.416).

## 4 Discussion

Our findings align with recent systematic comparisons showing that UBE can offer perioperative advantages over MD, such as reduced estimated blood loss and shorter hospital stay, while achieving comparable functional outcomes in pain and disability scores (21, 22). Unlike many previous analyses that focus on long-term outcomes only, this study elucidates clinical trajectories across early and intermediate postsurgical intervals, demonstrating statistically significant differences in early pain relief and recovery metrics. This temporal granularity is important for clinicians and patients when planning postoperative care and managing expectations.

Additionally, our emphasis on intervertebral space preservation provides new insights not emphasized in existing meta-analyses. While most comparative literature reports overall clinical parity between UBE and MD, few studies have systematically addressed anatomic outcomes that may influence spinal biomechanics and risk of adjacent segment degeneration. By documenting intervertebral height preservation over a six-month period, our results add a new dimension to assessments of surgical efficacy. Therefore, this study contributes not only to quantitative comparisons of clinical outcomes but also to a more nuanced understanding of how minimally invasive techniques may impact structural integrity post-discectomy.

The pathological essence of LDH is the protrusion of nucleus pulposus of lumbar intervertebral- disc or the damage of fibrous ring, which can cause the corresponding nerve roots to be compressed or stimulated, and induce the clinical manifestations of pain in waist and lower limbs, which is the key inducement to lose the ability to work and daily self-care (23, 24). The condition is frequently observed in individuals aged between 40 and 50 years old, with a lifetime incidence ranging from 13 to 40%. The occurrence of LDH is related to many factors, such as heredity, occupation, age, injury, degeneration, aseptic inflammation and so on (25, 26). Surgery emerges as the primary option for LDH when conservative treatments prove ineffective. Over time, the landscape of LDH surgical intervention has evolved from traditional open procedures towards minimally invasive techniques, with increasing emphasis placed on minimizing surgical trauma, perioperative discomfort, optimizing procedural precision, and facilitating swift postoperative recovery (26). Presently, minimally invasive surgical approaches for LDH, such as MSLD and UBE, are commonly employed. However, comprehensive research evaluating their specific efficacy and safety protocols remains scarce. Hence, this study aims to compare UBE and MSLD as surgical options for LDH.

The two surgical groups were well-matched at baseline and were analyzed patient-specific factors that may affect surgical outcomes. In line with previous literature, no significant outcome differences were observed between surgical levels L4–L5 and L5–S1 in our cohort, consistent with prior studies indicating comparable efficacy for these levels (27). Similarly, BMI did not affect postoperative improvement, aligning with evidence that obesity does not worsen pain relief or complication rates in lumbar discectomy. However, higher BMI may increase surgical complexity (28). Although bone mineral density (BMD) was not measured, its role is primarily relevant in fusion procedures. In decompression surgeries, BMD has less direct influence, though severely osteoporotic patients may have higher risk of postoperative vertebral body changes (29, 30). Overall, aside from slightly longer operative durations, obese and non-obese patients achieve comparable clinical outcomes with modern discectomy techniques.

UBE is a relatively newer evolution of endoscopic spinal surgery that utilizes two portals: one for visualization and the other for instrument access, facilitating broader manipulation using standard surgical tools. Though UBE involves two incisions and fluid irrigation, making it slightly less percutaneous than the uniportal method, it provides greater instrument flexibility and a more accessible learning curve for surgeons transitioning from microscopic techniques (31, 32). The clinical performance of UBE has been studied in comparison with both full-endoscopic and microscopic discectomy techniques (33). Multiple high-quality studies, including meta-analyses and randomized trials, have shown that UBE offers comparable clinical efficacy to MSLD in terms of pain relief, complication rates, and functional outcomes, while reducing soft tissue disruption and blood loss (34). We acknowledge the limitation regarding the sample size in this study. While we included a total of 80 participants (approximately 40 per group), which was sufficient for detecting differences in primary clinical outcomes such as pain relief, hospital stay, and recovery scores, it is possible that the sample size may have been underpowered to detect differences in low-frequency events such as complications. This could lead to Type II errors in detecting rare but important adverse outcomes (35).

Future studies should aim for larger sample sizes that can better capture the incidence of these less frequent events and ensure statistical power in assessing safety profiles and long-term complications. A sample size calculation should be performed in future studies to ensure adequate power to detect both primary and secondary outcomes, including rare complications. This will help to refine the clinical decision-making process and offer more robust evidence for the comparative safety of UBE versus MSLD in lumbar discectomy.

Our findings revealed that the operation time and total hospital stay in the EG were shorter, and the intraoperative blood loss was less. The VAS scores of the lower back and lower limbs in the EG were lower than those in the MG at 3 days, 3 months, and 6 months after operation, indicating that UBE was associated with shorter hospital stay, intraoperative blood loss, operation time and relieving postoperative pain. These findings accord with recent literature: a 2024 meta-analysis (9 studies; *n =* 1,001) likewise found UBE had less blood loss, shorter hospital stay and operative time, lower early back pain, and similar complications (36). In this study, all surgeries were performed by the same surgical team, which is a strength of our design in maintaining procedural consistency. However, as with any surgical technique, a learning curve may influence early outcomes. Specifically, it is possible that operative times and complication rates could have been higher in the earlier UBE cases as the surgical team became familiar with the technique. Several studies have documented that learning curve effects are inherent in the adoption of new surgical methods, particularly with minimally invasive approaches like UBE, which require precision and specific skill sets (37, 38).

Although our study did not specifically track operative times or complication rates across different stages of the learning process, it is important to note that the learning curve may have influenced early operative performance, particularly during the team’s initial experience with UBE. In future studies, it would be valuable to analyze operative times and complication rates separately for early and late cases to determine whether these factors affected the outcomes, and to account for any potential confounding associated with surgeon experience (39). This would allow for a more robust understanding of the impact of surgical expertise on the outcomes of UBE versus MSLD.

The advantages of UBE may stem from its small incision, which allows for separate observation and operation through two distinct independent channels in the affected area. These channels accommodate surgical instruments of conventional size, providing greater surgical flexibility and significantly enhanced efficiency (40, 41). By grinding off part of the lamina to expose ligamentum flavum, dura mater and nucleus pulposus, it can avoid the peeling of paravertebral muscles during operation, reduce the amount of bone removal and continuous perfusion, shorten the operation time, and reduce the amount of bleeding during operation (42, 43). The surgical incision of MSLD is large, and it is necessary to strip fascia, separate paraspinal muscles and remove some bony structures during the operation. The use of retractor for a long time may affect the blood circulation function, damage paraspinal muscles, affect the stability of the posterior structure of the spine, increase postoperative chronic pain and prolong the total hospitalization time (44, 45). Furthermore, administering continuous saline irrigation during a UBE procedure helps eliminate some inflammatory mediators and pain factors, hence mitigating postoperative discomfort after lumbar incision.

In addition to the observed benefits in surgical efficiency, the inflammatory prognosis of less invasive procedures like Unilateral Biportal Endoscopic (UBE) discectomy warrants consideration. Studies have shown that minimizing soft tissue disruption through smaller incisions and reduced muscle retraction can lead to lower levels of inflammatory cytokines and a faster recovery period postoperatively. Specifically, less invasive approaches are associated with a reduced inflammatory response, which can contribute to less postoperative pain and faster functional recovery. For instance, a recent study demonstrated that UBE discectomy leads to significantly lower serum levels of inflammatory markers such as C-reactive protein (CRP) and interleukin-6 (IL-6) when compared to traditional open surgical methods. This aligns with the notion that minimizing soft tissue trauma results in a more favorable inflammatory profile and faster recovery. Moreover, reducing the inflammatory burden may help to minimize long-term complications, such as chronic pain and delayed healing, which are often associated with more invasive surgeries (46).

The findings further demonstrated that the ODI index in the EG was lower than that of the MG on the 3rd day, 3rd month, and 6th month after operation, and the JOA score was higher in the EG. This pattern is broadly consistent with a meta-analysis, which found UBE and microdiscectomy had similar overall efficacy but lower back pain at 1–3 days and a small ODI advantage at final follow-up for UBE (no pooled ODI difference at 1–3 months), suggesting our 3-month ODI benefit is stronger than the pooled estimate while our 6-month advantage aligns with the reported “final follow-up” effect (36). The first multicenter randomized trial reported non-inferior 12-month ODI and slightly lower 48-h pain with UBE, indicating early pain benefits and comparable longer-term function (47). In comparison to MSLD, UBE may allow for more complete decompression of the spinal canal. Comparative LDH studies show similar functional outcomes at 12 months (and not clear superiority in “completeness” of decompression) when UBE is contrasted with microdiscectomy (48). The inclusion of laminectomy during the procedure aids in reducing pressure from ossification and hyperplasia of the ligamentum flavum, while also efficiently removing detached nucleus pulposus from the spinal canal (49, 50). Endoscopy in UBE can adjust the angle and enlarge the field of vision, reduce the damage to facet joints and prevent excessive resection. The procedure of MSLD involves the stripping of large paraspinal muscles and the incision of bilateral lamina and medial facet joints to reduce the stability of vertebral body and muscle after operation. During the operation, excessive traction on the nerve root and dural sac poses a risk of nerve root and dural sac damage, potentially affecting postoperative functional recovery (51, 52). Furthermore, it was observed that 6 months post-operation, the height of the diseased segmental intervertebral space in the EG was better preserved compared to that in the MG. UBE has shown superior preservation of intervertebral space height compared to MG in our study. Mechanistically, this might be attributed to the precision of the UBE technique, which involves minimal soft tissue disruption and reduced bone removal compared to traditional MSLD. The dual-portal approach of UBE allows for more targeted decompression with better visualization, potentially reducing the risk of damaging the surrounding structures, including the facet joints and ligaments, which are crucial for maintaining intervertebral stability. This targeted approach may help in maintaining the biomechanical integrity of the spine, particularly in the early postoperative period, where preserving disc height and structure is critical to long-term spinal health.

Furthermore, UBE’s ability to avoid excessive stripping of paraspinal muscles and its smaller surgical incision may reduce postoperative muscle atrophy and inflammation, both of which can contribute to the collapse of the intervertebral space after traditional surgeries. These factors, combined with less retraction of the nerve roots and reduced manipulation of the dura mater, might explain the better preservation of the intervertebral space in UBE, as the surrounding tissues and structures experience less trauma.

Endoscopic techniques do appear to better preserve structure: a 3-year study showed smaller loss of disc-height ratio after PELD than after MED, and UBE-specific imaging found larger bone-healing areas and higher bone-recovery ratios than microscopic discectomy (53). Together with a meta-analysis showing discectomy generally reduces disc height over time, our finding of better early disc-height preservation after UBE is plausible (54). This outcome supports the growing body of literature suggesting that UBE techniques, due to their precise decompression, minimal soft tissue disruption, and preservation of bony structures, result in superior anatomical preservation compared to MSLD (55, 56). Multiple studies have demonstrated that UBE causes less disruption to the intervertebral disc and surrounding structures, leading to less postoperative collapse of the disc space (57, 58). The use of continuous saline irrigation, limited muscle retraction, and endoscopic visualization may contribute to these structural advantages (59). This suggests that UBE may offer not only improved short-term recovery but also potential long-term biomechanical benefits by reducing postoperative disc collapse and preserving spinal segment stability.

There was no significant difference in the curative effect and the incidence of complications involving the two groupings 6 months after operation, which showed that the two methods could achieve good postoperative curative effect, and there were common complications of the two methods after operation. Preoperative motor weakness, present in a few patients in both groups, was comparable, but its impact on recovery was not analyzed. Literature suggests severe preoperative deficits may delay or limit strength recovery (60). Although there are differences in pain, functional recovery, and intervertebral space height between UBE and MSLD, after both types of surgical treatment, the above indexes significantly improved compared to preoperative levels. Both are minimally invasive operations with the same purpose. Patients can achieve good surgical results through accurate surgical positioning and professional operators, as well as professional postoperative nursing and rehabilitation training.

Table 7 shows no statistically significant difference in complication rates between the UBE and MSLD groups (*p* > 0.05), indicating that the current data cannot distinguish the safety of the two techniques. Although UBE was initially suggested to be safer, the lack of statistical significance means we cannot definitively conclude one is safer than the other. Larger studies with longer follow-up are needed to assess complications and safety profiles more accurately. Therefore, we should avoid overstating UBE’s safety based on the available evidence.

The complication rates between UBE and MSLD were comparable, with no statistically significant difference observed in our study. However, it is important to delve deeper into the specific complications encountered. Dural tears were observed in a small percentage of both groups, which aligns with the reported complication rates in the literature for both UBE and MSLD. Dural tears, while often not leading to major neurological deficits, can cause postoperative CSF leaks and prolonged recovery periods. The incidence of dural tears in our study was within the range of previously published data, which reports rates of approximately 2–5% for both MSLD and endoscopic procedures (61, 62). Similarly, instability was a concern in a few cases, though this was primarily seen in patients with pre-existing degeneration of the lumbar spine, and it did not lead to significant postoperative complications. The rate of instability in our cohort was comparable to the 3–4% reported in large series of lumbar discectomy (63). While UBE’s minimally invasive approach may reduce muscle and bone disruption, careful attention must still be paid to the possibility of inadvertent injury to the dura or nerve roots, especially when performing decompression at the lateral recess or near the foraminal space.

In terms of safety, while our study found no significant differences between UBE and MSLD in terms of complications, it is crucial to recognize that the relatively low complication rates might reflect the overall experience of the surgical team. Previous studies have noted that complications such as dural tears and instability can vary significantly based on the surgeon’s experience with the technique (64). This highlights the importance of training and technical proficiency in achieving favorable outcomes, particularly in complex cases.

Our study found that the intervertebral space height in the UBE group was better preserved compared to the MSLD group at 6 months post-operation. This finding is promising, suggesting potential benefits of UBE in preserving spinal biomechanics and minimizing long-term degeneration. However, we acknowledge that 6 months may not be sufficient to draw definitive conclusions about long-term biomechanical benefits, especially given the progressive nature of disc degeneration. While the early preservation of intervertebral space is an important metric, it is critical to recognize that long-term outcomes may differ as patients continue to recover and the spine undergoes natural aging processes and wear.

Additionally, measurement error is an important consideration when assessing intervertebral space height. While our study utilized X-ray imaging, which is standard in clinical practice, variability in measurements due to factors such as patient positioning and radiographic technique cannot be entirely excluded. We took steps to minimize error by ensuring that X-rays were performed under standardized conditions with consistent positioning. However, we must emphasize that radiographic measurements are inherently prone to small variations due to these factors, and future studies could benefit from employing more precise imaging techniques or utilizing three-dimensional imaging to enhance accuracy in assessing intervertebral space height and better control for measurement variability.

Given these considerations, we caution against drawing overly broad conclusions regarding the long-term biomechanical advantages of UBE based solely on 6-month data. While our findings are promising, they require further validation with longer follow-up periods, and future studies should incorporate more robust measurement controls and long-term tracking to determine the lasting effects of UBE on spine stability and intervertebral disc health.

UBE was associated with faster early recovery (lower blood loss, shorter stay, better early pain/function). At 6 months, modified MacNab outcomes were similar between groups. This does not establish equivalence; it indicates comparable overall success at 6 months on a four-category global scale, while early peri-operative advantages favor UBE.

In a single-team, standardized-pathway retrospective cohort of single-level LDH, UBE showed the above early advantages and better 6-month disc-height preservation. These observational findings are practice-relevant for centers with established UBE expertise: when early discharge and structural preservation are priorities, UBE may reasonably be selected as the first-line option, recognizing that short-term global success appears comparable to microdiscectomy. These data refine when UBE may be preferred, in experienced centers prioritizing early discharge and structural preservation, and delineate the outstanding questions (longer-term recurrence/reoperation, cost-effectiveness) for future trials. The key next step is a prospective, multicenter, preregistered non-inferiority trial of UBE versus microdiscectomy for single-level LDH. Power it on the 10-item ODI at 12–24 months, with leg-pain NRS/PASS, time-to-recurrence/reoperation, and cost-utility as core outcomes under standardized peri-operative care. Analyze with mixed-effects models that account for center and surgeon (learning curve), with prespecified multiplicity and transparent missing-data handling. If randomization is not feasible, a prospective registry using robust propensity methods and time-to-event models can still deliver decision-grade evidence.

We acknowledged certain limitations. First, as this was a retrospective, non-randomized study, potential selection bias and confounding (including confounding by indication) cannot be entirely excluded. Patients were grouped according to the surgical technique they had received rather than by random allocation, and certain clinical details may have been incompletely recorded, introducing information bias. In addition, potential confounders such as socioeconomic factors, surgeon preference, and patient comorbidities may have influenced treatment choice and outcomes, but were not fully controlled for in this analysis. Our 6-month follow-up captures early recovery but cannot evaluate long-term recurrence or stability. Prior studies report mean time-to-recurrence around 17–24 months (range 6–90 months), indicating that many recurrences occur beyond our observation window (65, 66). Therefore, future longitudinal studies with follow-up ≥12–24 months are necessary to evaluate long-term durability. Moreover, our study did not assess BMD, primarily because BMD has greater implications in fusion procedures rather than decompressive surgeries, though its role in long-term outcomes merits future exploration. Additionally, the retrospective design, lack of muscle strength and BMD assessments, and limited sample size preclude subgroup analyses by surgical level, BMI, or neurological status. Because modified MacNab is a course 4-level global rating, it may miss small differences that appear on continuous PROMs; therefore, similar MacNab proportions at 6 months should be read alongside the early continuous outcomes. Future prospective studies (≥12**–**24**-**month follow-up) with larger cohorts, and assessments of muscle strength and BMD are needed to evaluate sustained efficacy and long-term outcomes.

## 5 Conclusion

In conclusion, the findings of this study demonstrate that both UBE and MSLD exhibit favorable surgical outcomes in the treatment of LDH. While both procedures are associated with common postoperative complications and have a certain level of safety, UBE offers several advantages including reduced intraoperative bleeding, shorter operation duration, decreased postoperative pain, swifter functional recovery, and preservation of intervertebral space height. However, this study has some limitations. Continued investigations with larger patient cohorts, longer-term follow-up, and incorporation of additional objective measures will be valuable in further validating and refining these observations.

## Data Availability

The raw data supporting the conclusions of this article will be made available by the authors, without undue reservation.
